# Supplementary figures and images for: Thrombin Preconditioning of Extracellular Vesicles Derived from Mesenchymal Stem Cells Accelerates Cutaneous Wound Healing by Boosting Their Biogenesis and Enriching Cargo Content
Source: J Clin Med. 2019 Apr 18;8(4):533. doi: 10.3390/jcm8040533 (PMC6517934; doi:10.3390/jcm8040533)

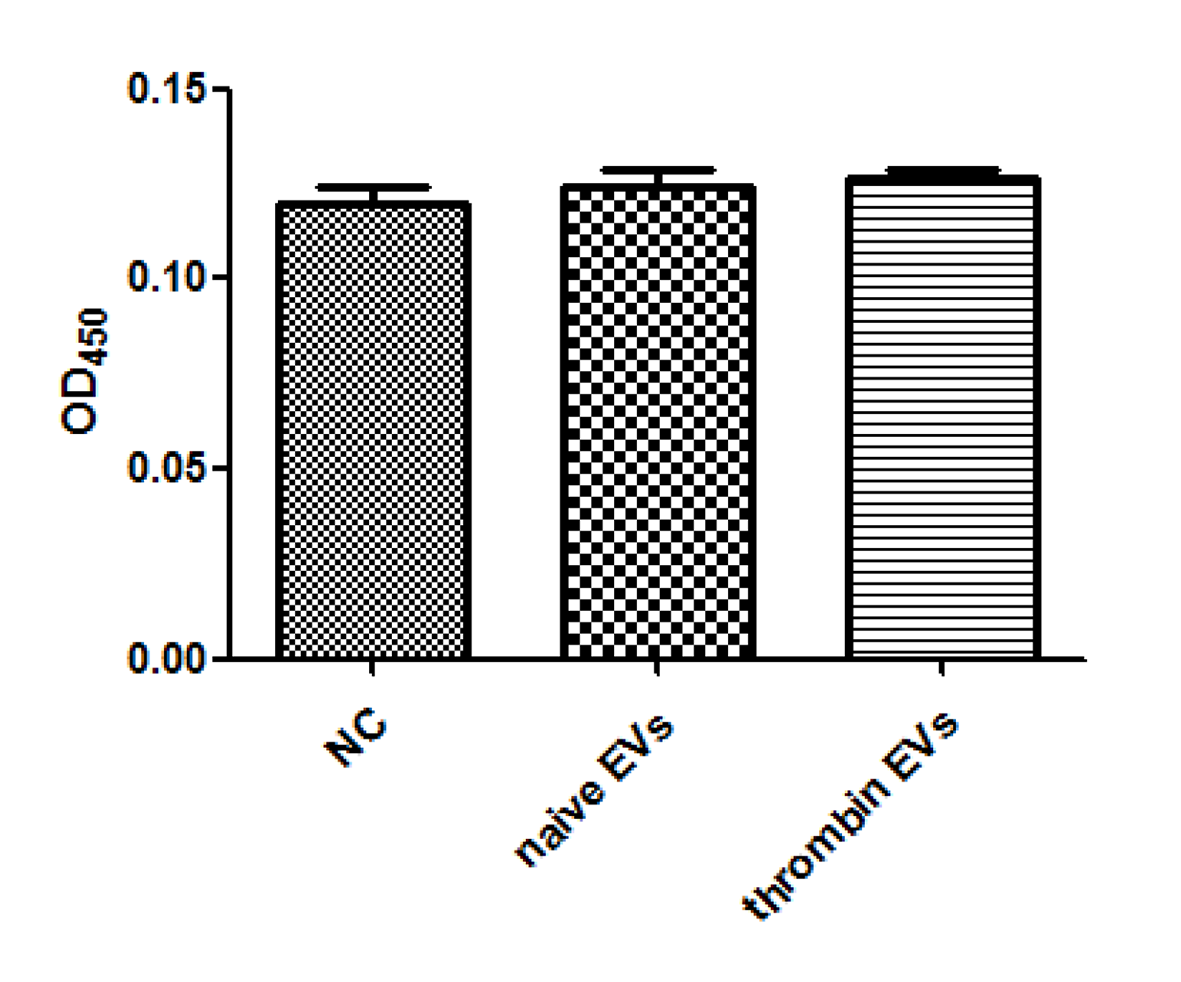

Supplement: Supplementary file 1 [file jcm-08-00533-s001.zip › jcm-468500-SI/jcm-468500-supplementary.tif]
